# Perivascular spaces and basilar artery remodeling in Fabry disease—a dual vascular pathology

**DOI:** 10.3389/fneur.2025.1689057

**Published:** 2025-12-15

**Authors:** Jieun Roh, Chong Kun Cheon, Soo-Yong Lee, Seung-Kug Baik, Min-Gyu Park, Kyung-Pil Park, Sung-Ho Ahn

**Affiliations:** 1 Department of Radiology, Research Institute for Convergence of Biomedical Science and Technology, Pusan National University Yangsan Hospital, Pusan National University School of Medicine, Busan, Republic of Korea; 2 Division of Medical Genetics and Metabolism, Department of Pediatrics, Pusan National University School of Medicine, Pusan National University Children's Hospital, Busan, Republic of Korea; 3 Department of Cardiology, Research Institute for Convergence of Biomedical Science and Technology, Pusan National University Yangsan Hospital, Pusan National University School of Medicine, Busan, Republic of Korea; 4 Department of Neurology, Research Institute for Convergence of Biomedical Science and Technology, Pusan National University Yangsan Hospital, Pusan National University School of Medicine, Busan, Republic of Korea

**Keywords:** Fabry disease, perivascular spaces, basilar artery, high-resolution vessel wall imaging, brain magnetic resonance imaging

## Abstract

**Background:**

Fabry disease (FD) is a lysosomal storage disorder that causes glycosphingolipid deposition in the vascular endothelium. Early neurovascular involvement is difficult to detect because conventional magnetic resonance imaging (MRI) findings overlap with age-related small- and large-vessel changes. We hypothesized that integrating microvascular and macrovascular MRI markers could improve the detection of FD-related vasculopathy.

**Methods:**

In a prospective case–control study, 26 genetically confirmed FD patients and 26 age- and sex-matched healthy controls underwent three-Tesla MRI (3 T MRI), including high-resolution vessel wall imaging. The macrovascular metrics included the basilar artery (BA) diameter, BA tortuosity index (BATI), and a composite BA degeneration index (BADI). The microvascular markers included the perivascular space (PVS) burden (Potter scale), white matter lesion severity (modified Fazekas scale), and global cerebral atrophy. Associations with FD were assessed using multivariable logistic regression, adjusting for age, sex, and vascular risk factors. Correlations between microvascular and macrovascular markers and age-stratified analyses were also performed.

**Results:**

Patients with FD exhibited a larger BA diameter, higher PVS burden in the basal ganglia and centrum semiovale, and greater cerebral atrophy than controls, while Fazekas scores were similar. Both PVS burden and BA diameter were independently associated with FD after adjustment, and the PVS burden remained significant after controlling for vascular risk factors. In patients with FD, but not in controls, the PVS burden correlated positively with the BADI, indicating coupled microvascular and macrovascular remodeling. Age-stratified analyses revealed steeper increases in BA metrics and PVS burden with advancing age in patients with FD, suggesting accelerated vascular degeneration.

**Conclusion:**

Combining the PVS burden with posterior circulation remodeling indices (BA diameter/BADI) reveals the disease-specific coupling of microvascular and macrovascular degeneration in FD. This quantitative MRI approach may enable earlier diagnosis, more precise risk stratification, and monitoring of therapeutic responses in clinical practice.

## Introduction

Fabry disease (FD) is a rare X-linked lysosomal storage disorder caused by an inherited deficiency of lysosomal *α*-galactosidase A, resulting from mutations in the *GLA* gene (1). This deficiency causes a progressive, systemic accumulation of globotriaosylceramide (Gb3) and its deacylated derivative, globotriaosylsphingosine (Lyso-Gb3), within lysosomes in various tissues, with a particularly pronounced impact on the vascular system (2). Consequently, endothelial dysfunction, microvascular disease, and inflammation are key contributors to organ damage in FD, affecting organs with dense microvascular networks (including the brain, heart, and kidneys) that are highly susceptible to vascular injury (3).

However, the heterogeneous clinical manifestations of FD and varying rates of disease progression make early diagnosis challenging (4, 5). Although microvascular involvement is a hallmark of FD, leading to small vessel disease in the kidneys (including proteinuria, decreased renal function, and end-stage renal disease), heart (including cardiomyopathy, arrhythmias, and heart failure), brain (including various forms of cerebral small-vessel disease), and skin (including angiokeratomas) (6), similar microvascular changes also commonly occur in the general aging population and are not unique to patients with FD. Similarly, macrovascular manifestations, which accelerate the progression of atherosclerosis and cause serious vascular complications such as coronary artery disease, ischemic or hemorrhagic strokes, and peripheral artery disease (7, 8), also occur frequently in older individuals and are not specific to patients with FD.

Given these limitations, we hypothesized that the combined assessment of microvascular and macrovascular degenerations could provide a more informative marker of FD-related vasculopathy than the examination of each type of vascular involvement separately. In this regard, brain magnetic resonance imaging (MRI), which can objectively and quantitatively evaluate both small- and large-vessel changes in the cerebral circulation, may be a valuable tool for characterizing FD-specific vasculopathy. Accordingly, we employed both conventional and high-resolution vessel wall imaging (HR-VWI), which offers several advantages, including enhanced spatial resolution, accurate assessment of vessel wall pathology, and better differentiation of disease processes, to investigate whether the combined assessment of cerebral microvascular and macrovascular changes could better characterize early vascular alterations in patients with FD.

## Methods

### Study patients

We prospectively enrolled patients aged ≥18 years with a genetic diagnosis of FD who were treated at Pusan National University Children’s Hospital.

A diagnosis of FD was confirmed by: (1) a pathogenic *GLA* gene mutation (in both men and women), and (2) decreased *α*-galactosidase A activity in leucocytes (in men) (9). The pathogenicity of the mutation was further supported by typical FD symptoms (including Fabry-specific neuropathic pain, angiokeratoma, or cornea verticillata in the patient or family members), elevated Lyso-Gb3 levels, biopsy of an affected organ showing characteristic zebra-body inclusions, or a previously reported pathogenic mutation. All patients were classified as having the classical or non-classical FD according to established criteria (10).

All participants provided informed consent for the study, including MRI examinations and the use of gadolinium-based contrast media (GBCA). This study was approved by the Institutional Review Board of the Pusan National University of Yangsan Hospital (IRB No.04–2020-018).

### Image protocols for HR-VWI and MRI

All patients underwent HR-VWI in addition to routine brain MRI using a 3 T scanner (Skyra; Siemens Healthineers, Enlargen, Germany) with a 64-channel head coil. The protocol included time-of-flight MR angiography, diffusion-weighted imaging, susceptibility-weighted imaging, fluid-attenuated inversion recovery (FLAIR), and HR-VWI using a Delay Alternating with Nutation for Tailored Excitation and Sampling Perfection with Application-optimized Contrast and Envelopes (DANTE-SPACE) sequence with proton-density and T2- and T1-weighted imaging (11). Contrast-enhanced imaging was performed only in individuals with normal renal function (serum creatinine <1.2 mg/dL and estimated glomerular filtration rate [eGFR] > 60 mL/min/1.73 m^2^).

The MRI findings in patients with FD were compared with those of age- and sex-matched controls who underwent both HR-VWI and routine brain MRI for headaches or possible extracranial cerebrovascular dissection. We focused on the differences in microvascular and macrovascular changes between patients with FD and controls and investigated the correlations between these two markers of cerebrovascular degeneration. The results of the imaging tests were independently evaluated by two specialized neuroradiologists in a blind manner, and in case of disagreement, a consensus was reached for the final decision.

### Image analysis for cerebral macrovascular degeneration

#### Diameter measurement of the internal carotid artery (ICA) and basilar artery (BA)

**Figure 1:**
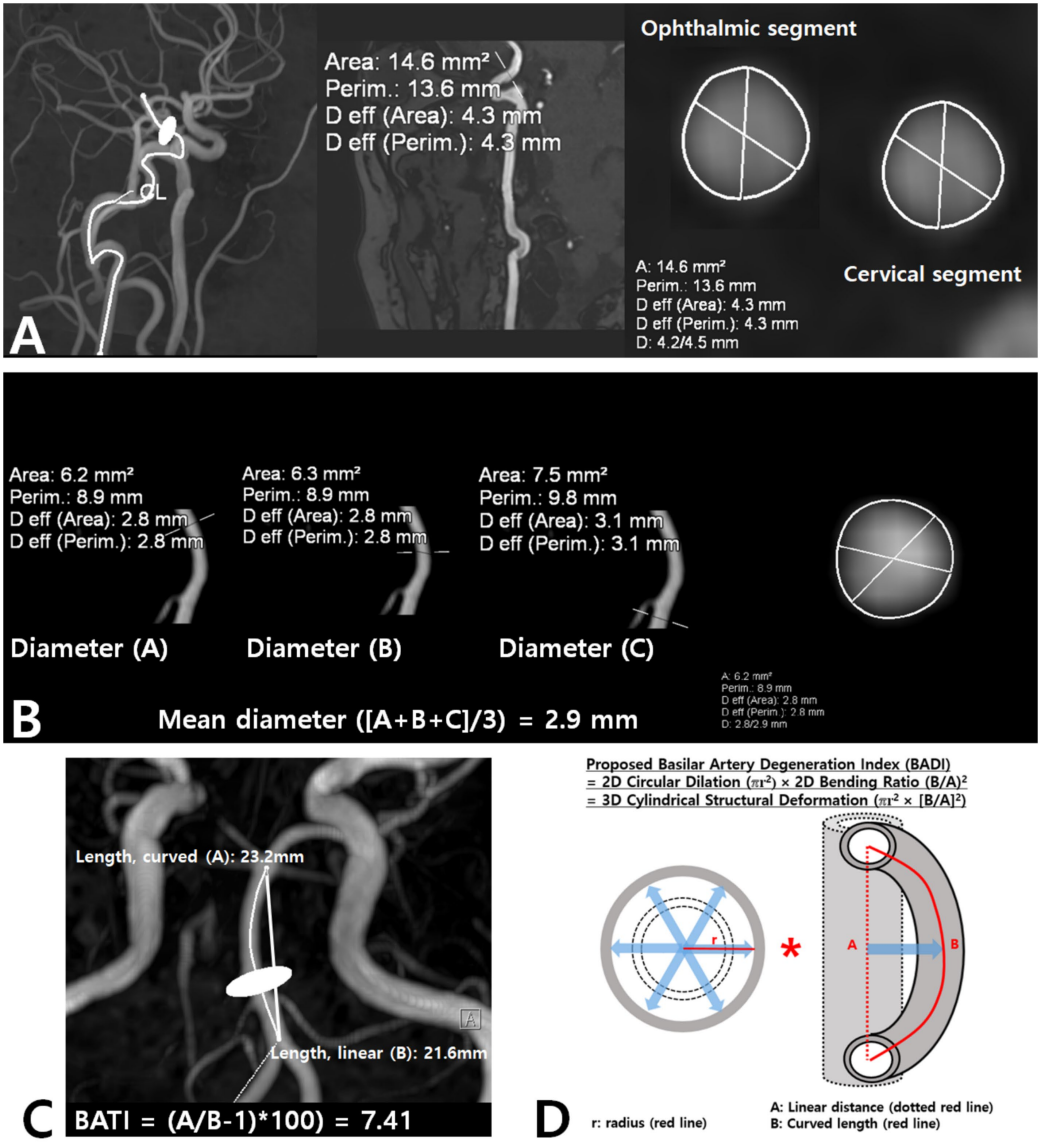
Assessment of ICA and BA diameters **(A,B)**, and measurement of BATI and BADI **(C,D)**. BA, basilar artery; BADI, basilar artery degeneration index; BATI, basilar artery tortuosity index; ICA, internal carotid artery.

ICA diameter was measured at both the cervical and intracranial segments, and the average value was calculated. BA diameter was measured at its caudal, intermediate, and rostral segments on axial images, and an average value was calculated (Figures 1A,B).

#### BA tortuosity measurement

The BA tortuosity index (BATI) was calculated as described previously (12), reflecting the ratio of the actual curved length of the BA to the straight-line distance between its proximal and distal endpoints, with higher values indicating greater tortuosity (Figure 1C).

#### Proposed concept of BA degeneration

We introduced the BA degeneration index (BADI) to capture the two-dimensional burden of BA degeneration, incorporating vessel diameter and tortuosity. The BADI was calculated as the product of the squared circular dilation πr^2^ and the squared cylindrical bending ratio (curved length/linear distance)^2^, as illustrated in Figure 1D.

### Image analysis for cerebral microvascular degeneration

#### Perivascular spaces (PVS)

**Figure 2:**
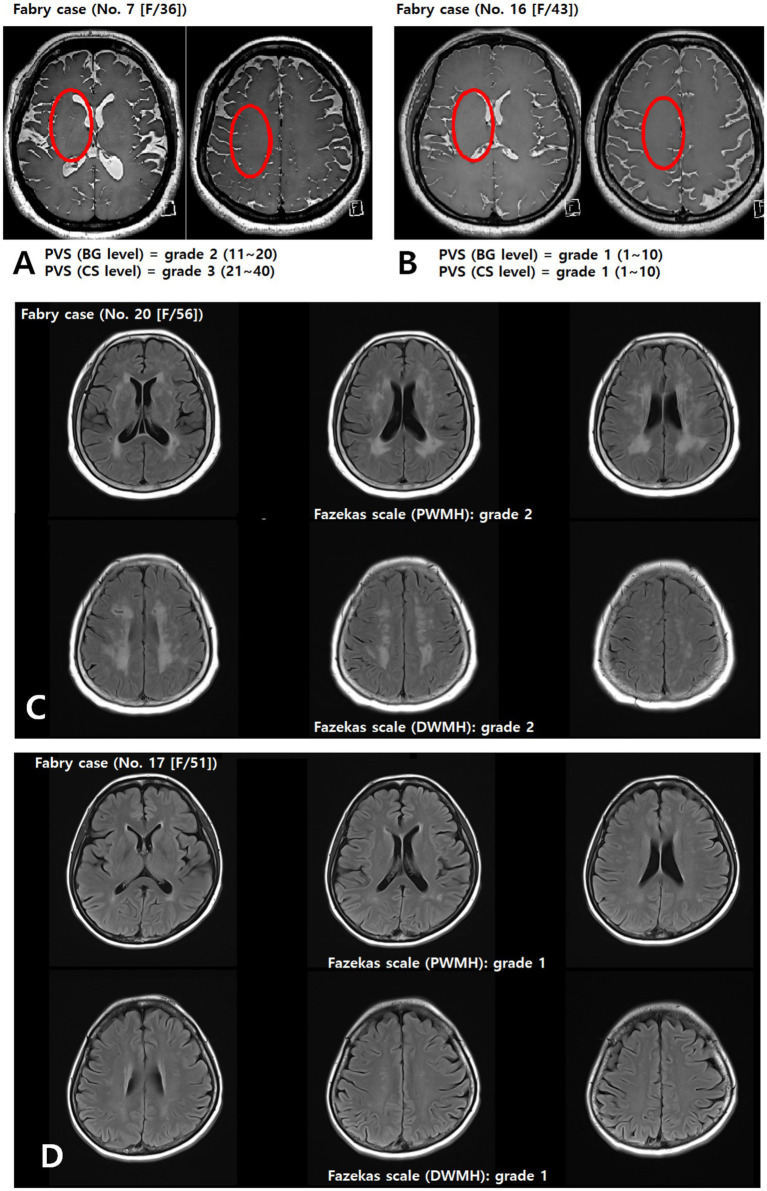
Assessment of the PVS **(A,B)** and Fazekas **(C,D)** scales. Representative axial T2-weighted image of a patient with FD showing multiple enlarged perivascular spaces (red circle) in the basal ganglia and centrum semiovale **(A)**. BG, basal ganglia; CS, centrum semiovale; DWMH, deep white matter hyperintensity; PVS, perivascular space; PWMH, periventricular hyperintensity.

PVS appear as round, oval, or linear lacunae with well-defined edges located in areas supplied by perforating arteries. They exhibit cerebrospinal fluid-like signal intensity (hypointense on T1-weighted images, hyperintense on T2-weighted images, and isointense on FLAIR images without a hyperintense rim). Although PVS are generally <3 mm in diameter, lesions ≥3 mm can be classified as PVS if they show a typical vascular morphology along the path of the perforating arteries. We used the Standards for Reporting Vascular Changes on Neuroimaging to evaluate the number, size (maximum diameter), and location of PVS (13). Thereafter, the overall PVS burden was rated using a visual scale adapted from Potter et al. (14) separately assessing the basal ganglia (BG) and centrum semiovale (CS) on each side of the brain. The final rating was the highest score in cases of asymmetry. Scores ranged 0–4 (0 = none, 1 = 1–10, 2 = 11–20, 3 = 21–40, 4 > 40), as shown in Figures 2A,B.

#### White matter lesions (WMLs)

WMLs were defined as hyperintensities on T2-weighted and FLAIR images, without cavitation. Severity was graded using a modified Fazekas scale (15), integrating periventricular hyperintensity (PWMH) and deep white matter hyperintensity (DWMH): 0 = absent; 1 = small, punctate foci (PWMH ≤ 10 mm and DWMH ≤ 10 mm); 2 = confluent or larger foci (DWMH ≤10 mm and PWMH ≥10 mm, or 10 mm ≤ DWMH < 25 mm, or DWMH ≥ 25 mm and PWMH < 10 mm); 3 = extensive confluent lesions (PWMH ≥ 10 mm and DWMH ≥ 25 mm), as shown in Figures 2C,D.

#### Cerebral atrophy

**Figure 3:**
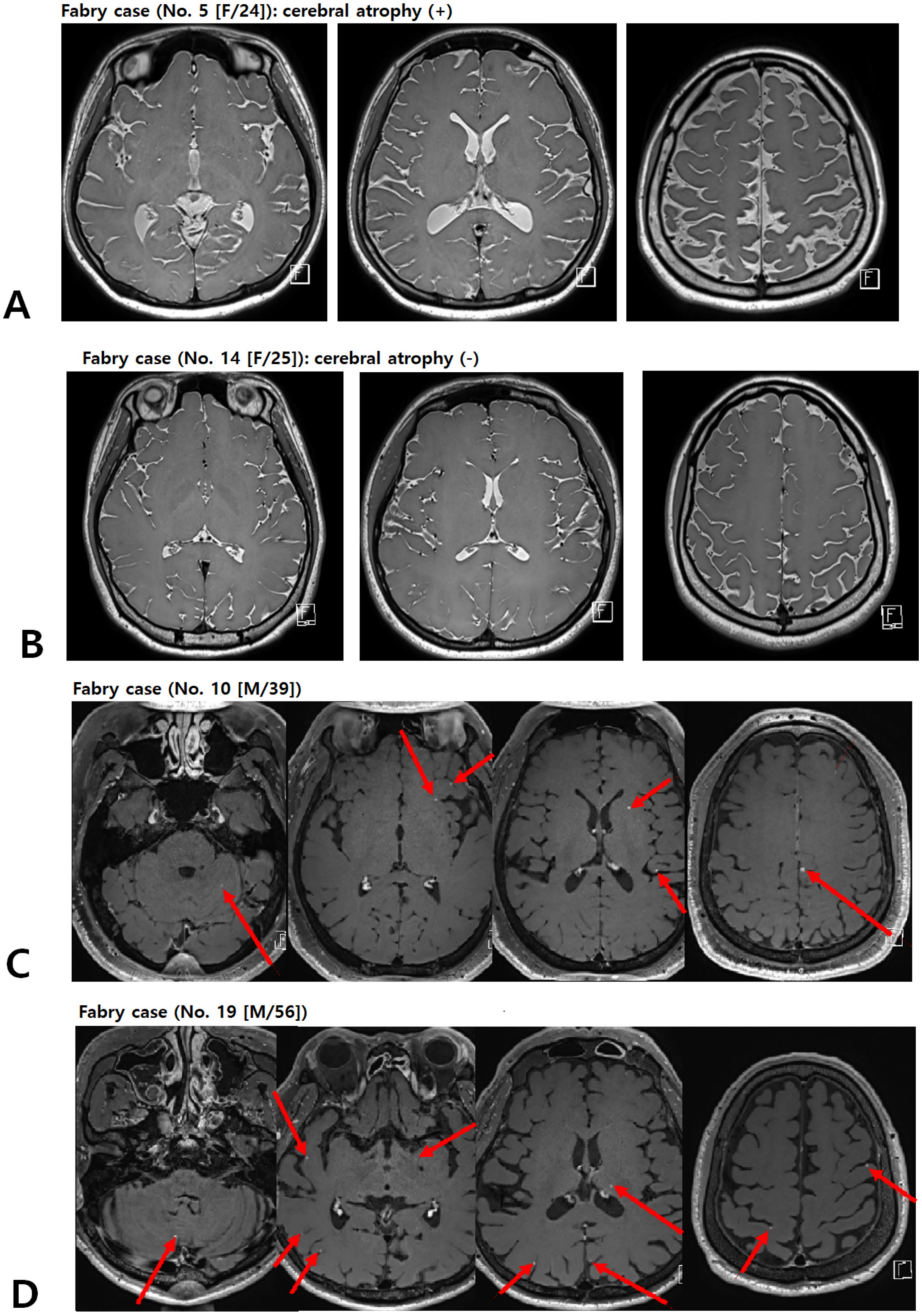
Representative picture of brain atrophy **(A,B)** and multiple enhancing lesions **(C,D)** in patients with FD. Contrast-enhanced T1-weighted images of patients with FD demonstrating multiple punctate/patchy parenchymal enhancing lesions (red arrows) (C and D). FD, Fabry disease.

Cerebral atrophy was graded using a semi-quantitative global cerebral atrophy scale: 0 = no atrophy (normal brain volume, no sulcal widening); 1 = mild atrophy (opening of sulci, early peripheral widening); 2 = moderate atrophy (gyral volume loss, diffuse widening of sulci); 3 = severe atrophy (severe gyral thinning, knife-edge appearance) (16). For analysis, grades 0 and 1 were combined as no/mild atrophy, and grades 2 and 3 were considered moderate-to-severe atrophy (Figures 3A,B).

### Clinical assessment

Demographics and atherosclerotic risk factors were documented, including hypertension (medication use or blood pressure >140/90 mm Hg on repeated measurements), diabetes mellitus (medication use, fasting glucose ≥126 mg/dL, or 2-h postprandial glucose ≥200 mg/dL), hyperlipidemia (fasting cholesterol >200 mg/dL or low-density lipoprotein ≥130 mg/dL), current or recent smoking, and renal insufficiency (eGFR <60 mL/min/1.73 m^2^ or receiving treatment for chronic kidney disease).

For FD-specific information, we recorded the age at diagnosis, age at treatment initiation, and duration of enzyme replacement therapy (ERT). Mutation types and baseline plasma lyso-Gb3 levels (measured using tandem mass spectrometry) were also recorded. Multisystem involvement (including neuropathic pain, ocular manifestations, skin lesions, and stroke history) was documented. Echocardiographic parameters included left ventricular mass index (LVMI) and left ventricular wall thickness (LVW), with the normal upper limits defined as LVMI ≤ 95 g/m^2^ for women and ≤ 115 g/m^2^ for men.

### Statistical analysis

Continuous variables are presented as mean ± SD or median (IQR), and categorical variables are presented as counts (%). Group comparisons were performed using Student’s *t*-test or Wilcoxon rank–sum test for continuous variables and Pearson’s *χ*^2^ or Fisher’s exact test for categorical variables, as appropriate.

Analyses were performed in two stages. First, to identify imaging markers associated with FD, we fitted logistic regression models for each candidate imaging variable that met a univariable screening threshold of *p* < 0.10. Effect sizes are presented as odds ratios (ORs) with 95% confidence intervals (CIs) per unit increase in the predictor (e.g., per 1-point increase in the PVS sum score and per 1-mm increase in the BA diameter). We prespecified two adjustment levels: a primary model adjusted for age and sex and a fully adjusted model additionally controlling for hypertension, diabetes, hyperlipidemia, current smoking, and renal insufficiency. Model assumptions were checked by inspecting the residuals and variance inflation factors, which were <5 in all final models.

In the second stage, after identifying imaging markers associated with FD in the first stage, we evaluated microvascular and macrovascular coupling among these key markers. Specifically, microvascular and macrovascular coupling was assessed using Spearman’s rank correlations among key FD-related microvascular and macrovascular indices in the total cohort and separately in the FD and control groups. We also calculated covariate-adjusted (partial) Spearman correlations using two prespecified covariate sets: a primary-adjusted set (age, sex, hypertension, diabetes, hyperlipidemia, smoking, renal disease, and Fazekas score) and a sensitivity-adjusted set (primary-adjusted plus cerebral atrophy), omitting Fazekas score or atrophy from the covariates when each was the target variable.

Multiple testing in the correlation and interaction analyses was controlled within each model block (total vs. subgroup; univariate vs. adjusted models) using the Benjamini–Hochberg false discovery rate (FDR) procedure with a target q = 0.05, and we report both raw *p*-values and FDR-adjusted *q*-values. To further examine whether these microvascular and macrovascular relationships differed by FD status, we additionally fitted linear regression models in the total cohort, with the FD-related microvascular marker identified in the first-stage analysis as the dependent variable and each imaging index, FD status, and their interaction term as independent variables, adjusted for the same covariates as in the primary-adjusted models.

A two-sided *p* < 0.05 was considered statistically significant before correction. Analyses were conducted using Statistical Package for Social Sciences (SPSS version 17.0) and R (version 4.x), as appropriate.

## Results

### Patients’ characteristics

Over 1 year, 26 patients with FD (12 classical and 14 non-classical) met the eligibility criteria. Of the 12 patients with classical FD (mean age 34.83 ± 13.85 years [range 21–72], five [41.7%] men), 10 received ERT for a mean duration of 7.2 years. Of the 14 non-classical patients with FD (mean age 51.64 ± 14.71 years [range 25–68], 2 [14.3%] men), eight received ERT for a mean duration of 5.9 years (Supplementary Table S1).

We ultimately included 52 participants (26 patients with FD and 26 age- and sex-matched controls), with a mean age of 43.71 ± 13.40 years (range, 21–72 years), of whom 14 (26.9%) were male. FD and control groups had similar overall demographics, except renal insufficiency, which was significantly more common in patients with FD (23.1% vs. 0%, *p* = 0.02). Consequently, six patients with FD underwent non-contrast MRI (Table 1).

**Table 1:** Comparisons between patients with fabry disease and healthy controls.

Variable	FD (*n =* 26)	Control (*n =* 26)	*p-*value^a^
Age (years)	43.88 ± 16.43	43.54 ± 9.81	0.93
Male	7 (26.9)	7 (26.9)	1.00
Medical history			
Hypertension	1 (3.8)	0 (0.0)	1.00
Diabetes mellitus	2 (7.7)	0 (0.0)	0.49
Hyperlipidemia	3 (11.5)	1 (3.8)	0.61
Current smoking	0 (0.0)	2 (7.7)	0.49
Renal insufficiency	6 (23.1)	0 (0.0)	0.02
Cerebral macrovascular degeneration			
Diameter of ICA, Right (mm)	3.996 ± 0.454	3.771 ± 0.385	0.60
Diameter of ICA, Left (mm)	4.023 ± 0.380	4.127 ± 0.426	0.36
Diameter of BA, mean (mm)	3.177 ± 0.413	2.962 ± 0.283	0.03
BATI	9.437 ± 5.705	8.655 ± 5.135	0.62
BADI	9.788 ± 4.016	8.224 ± 1.746	0.07
Cerebral microvascular degeneration			
PVS score, sum	4 [3–5]	3 [2–3.25]	< 0.01
BG level	2 [1–2]	1 [1–1.25]	< 0.01
CS level	2 [2–3]	2 [1–2]	< 0.01
Fazekas scale, sum	0 [0–1.25]	0 [0–1.25]	0.43
PWMH	0 [0–0.25]	0 [0–1.00]	0.15
DWMH	0 [0–1.00]	0 [0–1.00]	0.53
Cerebral atrophy	11 (42.3)	0 (0.0)	< 0.01

Variables are presented as mean ± standard deviation, median (interquartile range), or numbers (%). BA, basilar artery; BADI, basilar artery degeneration index; BATI, basilar artery tortuosity index; BG, basal ganglia; CS, centrum semiovale; DWMH, deep white matter hyperintensity; FD, Fabry disease; ICA, internal carotid artery; PVS, perivascular space; PWMH, periventricular hyperintensity. ^a^*p*-values were calculated using Pearson’s chi-square test, Fisher’s exact test, Student’s *t*-test, or Wilcoxon rank–sum test as appropriate.

### Analysis for cerebral microvascular and macrovascular degeneration

As shown in Table 1, patients with FD exhibited a larger mean BA diameter (3.177 ± 0.413 mm vs. 2.962 ± 0.283 mm) and a trend toward a higher BADI (9.788 ± 4.016 vs. 8.224 ± 1.746) than the controls.

Regarding microvascular markers, patients with FD had a higher median PVS score (4 (3–5) vs. 3 [2–3.25]) in both the BG and CS regions than controls. In contrast, the mean Fazekas scores were not significantly different. The prevalence of cerebral atrophy was significantly higher in patients with FD (11 [42.3%] vs. 0 [0.0%]) than in controls.

### Factors associated with FD

In the multivariable logistic regression analysis (Table 2), the PVS sum score (OR = 4.638, 95% CI: 1.912–11.253) and BA diameter (OR = 7.635, 95% CI: 1.039–56.126) were independently associated with FD after adjusting for age and sex. Moreover, the PVS score remained significantly associated with FD (OR = 5.307, 95% CI: 1.734–14.634) even after adjusting for age, sex, and other vascular risk factors. Given this robust association, the PVS burden was treated as the primary microvascular marker in subsequent microvascular and macrovascular coupling analyses.

**Table 2:** Multivariable logistic regression analysis of the association between Fabry disease and radiographic markers.

Variables	Univariate			Multivariate^a^			Multivariate^b^		
	OR	95% CI	*p*-value	OR	95% CI	*p*-value	OR	95% CI	*p*-value
PVS score, sum	3.141	1.592–6.196	<0.01	4.638	1.912–11.253	<0.01	5.037	1.734–14.634	<0.01
Diameter of BA, mean (mm)	7.581	1.021–56.261	0.05	7.635	1.039–56.126	0.05	7.134	0.569–89.507	0.13
BADI	1.362	0.962–1.930	0.08	1.363	0.963–1.931	0.08	1.296	0.849–1.977	0.23

BA, basilar artery; BADI, basilar artery degeneration index; CI, confidence interval; FD, Fabry disease; OR, odds ratio; PVS, perivascular space. ^a^Adjusted for age and sex. ^b^Adjusted for age, sex, and vascular risk factors, including hypertension, diabetes mellitus, hyperlipidemia, current smoking, and renal insufficiency.

### Interaction between cerebral microvascular and macrovascular degenerations

Given that the PVS burden emerged as the FD-related microvascular marker in the multivariable models (Table 2), we next examined its relationships with macrovascular and parenchymal indices. In the total cohort, higher PVS scores showed modest positive correlations with BADI (*ρ* = 0.345; *p* = 0.012; q = 0.085), age, and ICA diameter in unadjusted analyses. Among the tested microvascular and macrovascular pairs, the PVS–BADI relationship was the only association that remained statistically significant in both the primary- and sensitivity-adjusted models before multiple-testing correction, but none of these associations survived FDR adjustment. In the FD subgroup, the PVS–BADI coupling was numerically stronger (unadjusted *ρ* = 0.537; *p* = 0.0047; q = 0.033) and remained directionally consistent after covariate adjustment (primary-adjusted *ρ* = 0.427; *p* = 0.0297; q = 0.208; sensitivity-adjusted *ρ* = 0.391; *p* = 0.0484; q = 0.339), yet again failing to meet the FDR threshold (Table 3).

**Table 3:** Correlations between the FD-related key marker (PVS burden) and microvascular and macrovascular imaging indices in the total cohort and by FD status.

Variable	Univariate			Primary adjusted			Sensitivity adjusted		
	*ρ*	*p*-value	q (FDR)	*ρ*	*p*-value	q (FDR)	*ρ*	*p*-value	q (FDR)
(1) Total cohort									
Diameter of ICA, Right	0.306	0.0275	0.0964	0.103	0.4661	0.4661	0.041	0.7739	0.7739
Diameter of ICA, Left	0.031	0.8274	0.8274	−0.105	0.4584	0.5347	−0.088	0.5331	0.6220
Diameter of BA	0.245	0.0803	0.1873	0.172	0.2224	0.5189	0.121	0.3942	0.6899
BATI	0.164	0.2448	0.2856	0.134	0.3436	0.6012	0.137	0.3318	0.7741
BADI	0.345	0.0122*	0.0851	0.303	0.0287*	0.2011	0.269	0.0539	0.3776
Fazekas, sum	0.216	0.1241	0.1738	−0.106	0.4557	0.6380	−0.105	0.4576	0.6406
Cerebral atrophy	0.239	0.0880	0.1541	0.186	0.1869	0.6542	0.186	0.1869	0.6542
(2) FD cohort									
Diameter of ICA, Right	0.248	0.2220	0.3885	0.114	0.5808	0.6776	0.071	0.7312	0.8531
Diameter of ICA, Left	0.234	0.2503	0.3504	0.120	0.5603	0.9806	0.117	0.5683	0.9946
Diameter of BA	0.398	0.0442*	0.1031	0.212	0.2985	1.0000	0.175	0.3933	1.0000
BATI	0.079	0.7020	0.8190	0.118	0.5671	0.7940	0.106	0.6075	0.8505
BADI	0.537	0.0047*	0.0330	0.427	0.0297*	0.2079	0.391	0.0484*	0.3388
Fazekas, sum	0.409	0.0383	0.1339	0.140	0.4965	1.0000	0.119	0.5614	1.0000
Cerebral atrophy	−0.005	0.9791	0.9791	0.012	0.9550	0.9550	0.012	0.9550	0.9550
(3) Control cohort									
Diameter of ICA, Right	0.204	0.3164	1.0000	0.275	0.1746	1.0000	0.275	0.1746	1.0000
Diameter of ICA, Left	0.079	0.7028	0.8434	−0.085	0.6804	0.8165	−0.085	0.6804	0.8165
Diameter of BA	−0.162	0.4293	0.6439	−0.131	0.5236	0.7854	−0.131	0.5236	0.7854
BATI	0.187	0.3600	1.0000	0.215	0.2913	0.8738	0.215	0.2913	0.8738
BADI	−0.006	0.9772	0.9772	0.143	0.4850	0.9699	0.143	0.4850	0.9699
Fazekas, sum	0.162	0.4282	0.8564	−0.050	0.8072	0.8072	−0.050	0.8072	0.8072
Cerebral atrophy	N/A	N/A	N/A	N/A	N/A	N/A	N/A	N/A	N/A

Primary-adjusted model: age, sex, hypertension, diabetes, hyperlipidemia, current smoking, renal disease, and Fazekas score were adjusted. Sensitivity-adjusted model: same covariates plus cerebral atrophy. When the target variable was the Fazekas score or cerebral atrophy, that marker was excluded from the covariates. N/A indicates a constant variable or an effective sample size of <10. The false discovery rate (FDR; *q* = 0.05, Benjamini–Hochberg) was controlled for each model block. *p*-values were derived from Spearman’s rank correlation; q-values represent false discovery rate–adjusted *p*-values using the Benjamini–Hochberg method within each model block. Asterisk (*) indicates adjusted *p*-value *q* < 0.05 within each model block, and bold indicates FDR-adjusted *q* < 0.05. FD, Fabry disease; ICA, internal carotid artery; BA, basilar artery; BATI, basilar artery tortuosity index; BADI, basilar artery degeneration index; PVS, perivascular spaces.

In prespecified interaction analyses using linear regression models with the FD-related microvascular marker identified in the first-stage analysis (PVS sum) as the dependent variable, each vascular or parenchymal imaging index (ICA diameter, right/left; BA diameter; BATI; BADI; Fazekas score; and cerebral atrophy), FD status, and their interaction term were entered, with adjustment for age, sex, and vascular risk factors. No interaction term reached statistical significance after FDR correction (all q ≈ 0.97; Supplementary Table S2), indicating that the slopes of the associations between PVS burden and imaging indices did not differ materially between the FD and control groups.

### Age-specific distribution of cerebral microvascular and macrovascular degenerations

**Figure 4:**
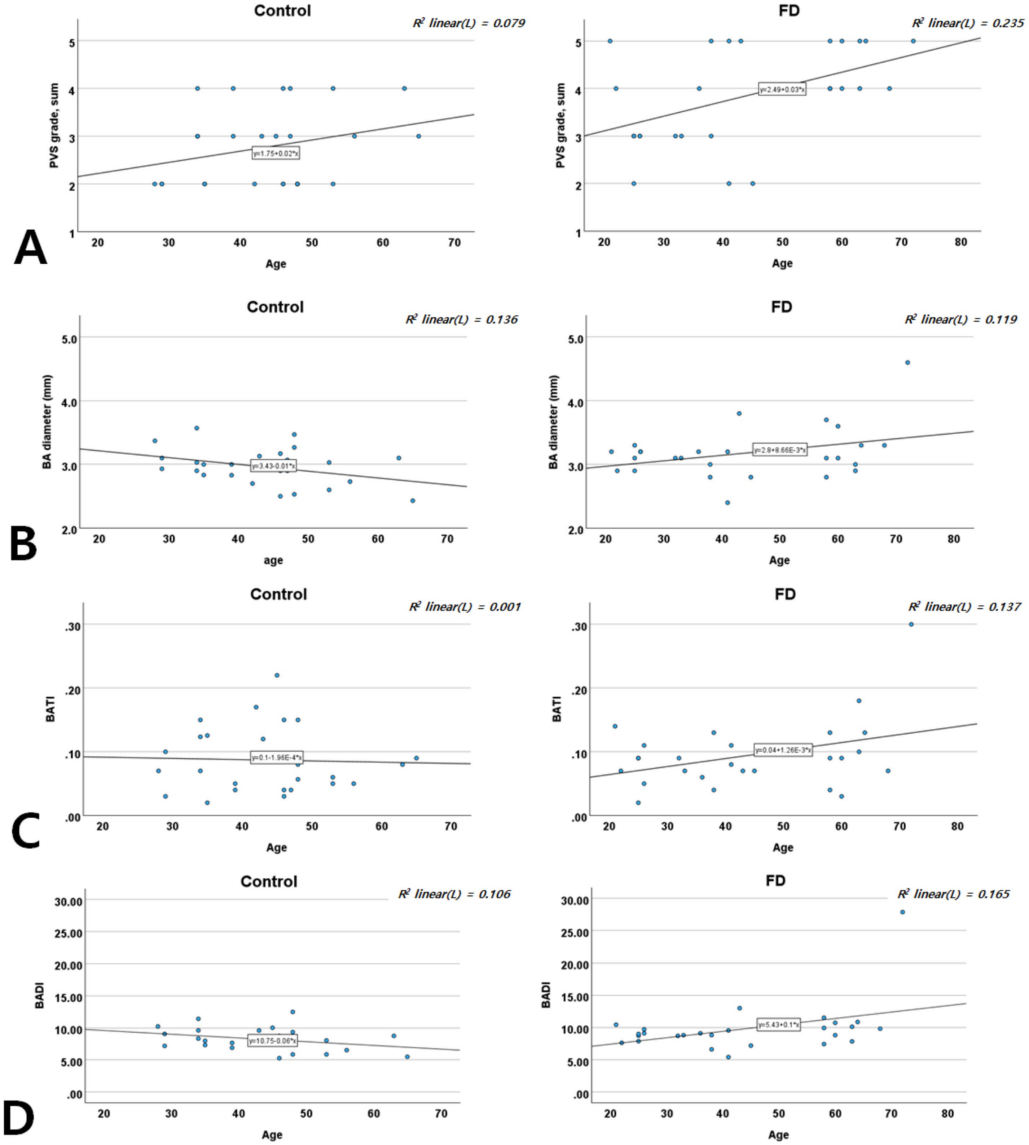
Age-specific distribution of the PVS score **(A)**, BA diameter **(B)**, BATI **(C)**, and BADI **(D)** in patients with FD and controls. BA, basilar artery; BADI, basilar artery degeneration index; BATI, basilar artery tortuosity index; FD, Fabry disease; PVS, perivascular space.

In both groups, the PVS scores increased with age. However, increases in BA diameter, BATI, and BADI with advancing age were observed only in patients with FD, not in controls (Figure 4), suggesting an accelerated pattern of vascular degeneration in patients with FD.

## Discussion

In this study, we investigated whether combining microvascular and macrovascular neuroimaging markers could better characterize FD-related cerebrovascular involvement and potentially facilitate early recognition of FD-related vasculopathy. Using HR-VWI and conventional MRI, we assessed ICA and BA diameters, BATI, and our proposed BADI as macrovascular metrics, alongside PVS, WMLs (modified Fazekas score), and cerebral atrophy as microvascular markers.

Patients with FD showed significantly higher PVS scores and a greater prevalence of cerebral atrophy, indicating small-vessel involvement and prominent macrovascular changes in the posterior circulation, including a larger BA diameter and degenerative changes. These findings are consistent with the pathological accumulation of Gb3 and Lyso-Gb3 in the vascular endothelium, affecting both large and small vessels of the heart. Notably, PVS scores correlated with BADI, suggesting a shared pathophysiological mechanism linking microvascular and macrovascular degeneration in FD (17, 18). Age-stratified analyses further revealed a steeper trajectory of vascular deterioration in FD than in controls, implying that glycosphingolipid accumulation accelerates vascular aging and structural damage (19).

FD is known to confer a high burden of cerebrovascular complications, including transient ischemic attacks and stroke (affecting nearly one-quarter of patients at a mean onset age of approximately 40 years) (20, 21), vertebrobasilar dolichoectasia (22, 23), and early, diffuse cerebral small-vessel disease and cerebral atrophy (24, 25). Although these findings likely reflect accelerated microvascular (white matter lesions, lacunar infarctions, and increased PVS) and macrovascular (vertebrobasilar dolichoectasia) changes in FD (26, 27), similar changes are also frequently observed in older individuals with conventional vascular risk factors, complicating FD-specific diagnosis (28).

**Figure 5:**
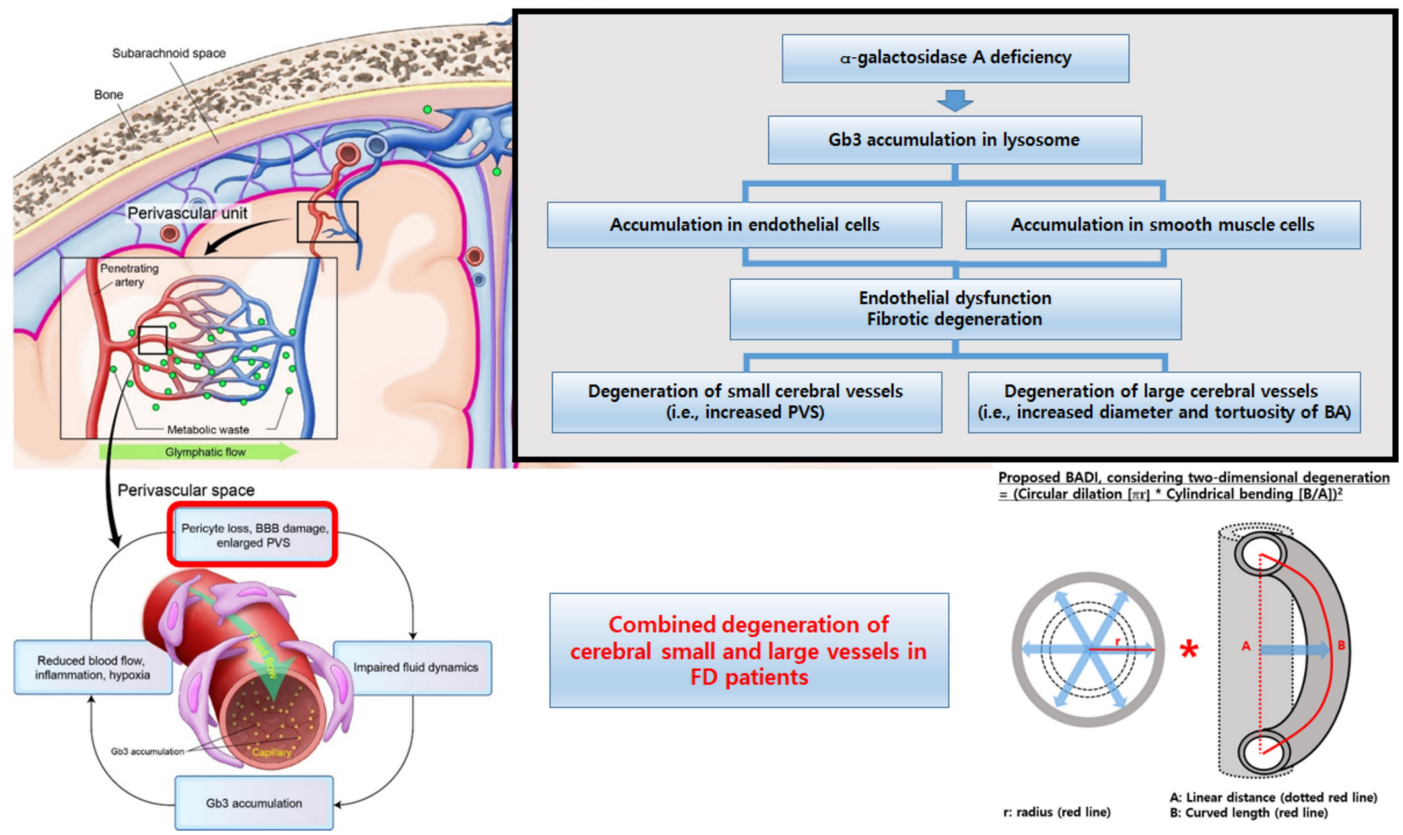
Conceptual summary of cerebral vascular involvement in Fabry disease. Schematic illustration showing the combined microvascular (increased PVS burden and cerebral atrophy) and macrovascular (enlarged and tortuous basilar artery, higher BADI) changes in Fabry disease and their proposed coupling and age-accelerated progression compared with those in the controls. BA, basilar artery; BADI, basilar artery degeneration index; BBB, blood–brain barrier; FD, Fabry disease; PVS, perivascular space.

Collectively, our findings support a systemic pattern of vascular involvement in FD, affecting both small and large cerebral vessels (Figure 5). Early integrated MRI assessment of these markers may help identify patients at a higher cerebrovascular risk and refine the phenotypic characterization of FD-related vasculopathy. However, these measures should currently be regarded as candidate imaging markers rather than validated diagnostic tools, and larger longitudinal studies are needed to define their diagnostic accuracies and clinical utilities.

### Cerebral microvascular degeneration: PVS in FD

Our data indicate that patients with FD had a significantly higher PVS burden than did the controls, and that this metric was independently associated with FD status, even though the groups did not differ substantially in WMLs severity (modified Fazekas score). PVS are fluid-filled spaces surrounding penetrating arteries (29), typically inconspicuous on MRI unless there is microvascular dysfunction, increased interstitial fluid, or impaired perivascular drainage (30). Although PVS enlargement can be influenced by aging, vascular risk factors (including hypertension), or degenerative central nervous system diseases (31), the higher PVS burden in patients with FD suggests an FD-specific process, likely involving neuroinflammatory mechanisms, glycosphingolipid accumulation in small vessels, and disruption of vascular homeostasis (32).

Interestingly, although the modified Fazekas score was not significantly different between patients with FD and controls, the PVS scores were distinctly elevated in patients with FD. This discrepancy suggests that PVS may detect subtle microvascular stress at earlier stages of the disease. Clinically, an increased PVS burden may precede the development of larger WMLs or lacunar infarcts (33), positioning PVS as a potentially sensitive imaging biomarker for subclinical vascular damage (34). This is especially pertinent in FD, where neurological complications often present at a younger age than in the general population. Moreover, our observation that patients with FD show higher rates of cerebral atrophy, even with relatively modest WMLs, underscores the subtle but pervasive nature of FD-related microvascular compromise, which can lead to neuronal and axonal loss and subsequent neurodegeneration over time.

### Cerebral macrovascular degeneration: BA metrics in FD

In parallel with microvascular changes, patients with FD exhibit significant macrovascular abnormalities, particularly in the posterior circulation. We observed increased BA diameter, tortuosity (BATI), and degeneration (BADI), which are consistent with arterial remodeling or dolichoectasia. Previous studies have similarly reported intracranial arterial dilation and dolichoectasia in FD, where glycosphingolipid deposition may weaken vessel walls, promote fibrosis, and spur compensatory remodeling (22, 35).

From a clinical standpoint, detecting such structural changes in major arteries could be crucial for risk stratification, even in the absence of overt aneurysms or stroke. Subclinical macrovascular changes may alter local hemodynamics and cause compressive symptoms or embolic events, especially in the posterior circulation. Standard MRI or carotid duplex imaging, which often emphasizes stenosis, may overlook arterial dilation and tortuosity. Thus, measuring BA diameter, tortuosity, and the integrated degenerative index (BADI) could be central to uncovering subclinical macrovascular compromise in patients with FD. These indices may also serve as quantitative markers for monitoring disease progression and response to therapy.

### Combined microvascular and macrovascular degenerations: PVS and BADI in FD

A key finding of our study was the moderate positive correlation between PVS scores and BADI in patients with FD but not in the controls. This points to a disease-specific process in which glycosphingolipid accumulation, chronic inflammation, and endothelial dysfunction simultaneously affect vessels of different calibers (36). Dolichoectasia in large vessels may adversely affect hemodynamics in smaller arterioles, contributing to a higher PVS burden, and vice versa, as widespread microvascular involvement can exacerbate flow dysregulation in larger vessels (17, 26, 27).

Clinically, this observation suggests that pronounced microvascular changes on MRI (including elevated PVS) should prompt the evaluation of the BA for early ectatic or tortuous changes in the BA. Conversely, dolichoectatic changes in the posterior circulation should prompt a scrutiny of microvascular pathology. This two-pronged approach could refine risk stratification and aid in treatment optimization, including the timely initiation or intensification of ERT or chaperone therapy. Longitudinal studies are needed to confirm whether improvements in the PVS burden and macrovascular indices (including BADI) parallel better outcomes or reduced stroke risk.

### Age-specific distribution of neuroimaging markers

Our age-stratified analyses showed that while both patients with FD and controls exhibited increased PVS scores with age, patients with FD experienced steeper increases in PVS and BA metrics (diameter, BATI, and BADI). This suggests that FD accelerates typical age-related vascular changes through the additive effects of Gb3 and Lyso-Gb3 deposition. This accelerated vascular aging may explain the higher incidence of stroke and cognitive impairment at younger ages in FD (37, 38).

These findings support the need for earlier and more frequent imaging surveillance for FD. Advanced imaging might be reserved for later decades in individuals without FD, whereas patients with FD could benefit from more proactive, earlier screening to identify high-risk individuals who might require intensified therapeutic interventions. Moreover, characterizing these age-specific trajectories could help pinpoint critical windows for intervention, such as the optimal timing for ERT escalation (5).

### Abnormal enhancement pattern in FD

An incidental yet clinically relevant finding was the presence of multiple punctate or patchy enhancing lesions in approximately half of the patients with FD who underwent contrast-enhanced MRI (9 of 20 patients; Figures 3C,D). These lesions were not part of our *a priori* imaging endpoints but were identified during a systematic review of post-contrast T1-weighted sequences. In all cases, additional systemic imaging (including chest and abdominal CT, and, when indicated, PET-CT) and repeat contrast-enhanced brain MRI at 3–6 months failed to reveal evidence of an underlying malignancy, and the lesions remained stable or regressed over time.

Although histopathological confirmation was not available, the morphology and distribution of these punctate or curvilinear enhancements are compatible with small-vessel or perivascular involvement and may reflect, at least in part, blood–brain barrier (BBB) disturbance rather than neoplastic disease, in line with previous descriptions of similar enhancing patterns in small-vessel and inflammatory disorders (39).

In the context of FD, potential mechanisms include Gb3 accumulation in endothelial and smooth muscle cells, chronic endothelial dysfunction and inflammation, and altered local hemodynamics in ectatic or tortuous vessels, all of which could increase BBB permeability. However, these findings should be interpreted cautiously and viewed as hypothesis-generating; dedicated prospective studies using standardized contrast-enhanced MRI and quantitative BBB imaging techniques will be required to determine the prevalence, specificity, and prognostic significance of such lesions in FD (40).

## Limitations

This study had several limitations. First, the sample size was small (26 patients with FD and 26 controls), limiting generalizability and statistical power; larger cohorts are required to validate these findings. Second, the control group comprised individuals undergoing MRI for headache or possible extracranial cerebrovascular dissection, which may not fully represent the general population and may have introduced selection bias. Third, the study was cross-sectional, preventing inferences about causality or disease progression; longitudinal studies are required to clarify how vascular changes evolve over time. Fourth, imaging modalities such as HR-VWI may be influenced by technical factors, and subjective image interpretation may affect measurement reproducibility. Fifth, the control group was not genetically screened for FD; therefore, the presence of undiagnosed FD carriers cannot be completely ruled out. Sixth, although atherosclerotic risk factors (including hypertension and diabetes) were recorded, they were not rigorously controlled or modelled, and more advanced statistical approaches in larger datasets could further clarify the independent effect of FD. Seventh, we relied on PVS as our primary microvascular marker; additional modalities, such as diffusion tensor imaging or cerebral blood flow analysis, might provide complementary information. Eighth, many patients with FD were receiving enzyme replacement therapy, but the impact of treatment on cerebrovascular imaging markers was not thoroughly examined, and the study was not powered for detailed ERT- or phenotype-stratified analyses. Finally, more extensive data on disease severity, mutation types, and longitudinal clinical outcomes would enhance the understanding of FD-related vascular involvement, and stratifying results by FD subtype (classical vs. non-classical) could yield more targeted insights. Ongoing longitudinal follow-up (3–5 years) with repeated high-resolution imaging is expected to clarify how these vascular markers evolve according to the FD phenotype and ERT exposure.

In addition, the punctate or patchy enhancing lesions observed in a subset of patients with FD were identified in an exploratory analysis of clinically indicated contrast-enhanced MRI rather than as a prespecified endpoint and were not confirmed histopathologically. Therefore, these findings should be interpreted cautiously and warrant confirmation in dedicated prospective studies with standardized contrast-enhanced protocols.

## Conclusion

This study highlights the value of integrating microvascular and macrovascular MRI markers to characterize early cerebrovascular degeneration in FD, showing that patients with FD have an increased PVS burden, more cerebral atrophy, and prominent macrovascular changes in the posterior circulation. PVS and BA degeneration were moderately correlated, suggesting a shared pathophysiological mechanism driven by Gb3 and Lyso-Gb3 deposition. By employing advanced MRI techniques, such as high-resolution vessel wall imaging, subtle vascular alterations can be identified earlier, offering promising candidate biomarkers for vascular risk stratification and monitoring rather than definitive diagnosis. Ultimately, this integrative imaging approach may support personalized management strategies, such as optimized enzyme replacement therapy and adjunctive interventions, to prevent or mitigate vascular injury in patients with FD. Larger longitudinal and treatment-stratified studies are needed to validate these imaging markers and define the best way to incorporate them into routine clinical screening and follow-up.

## Data Availability

The raw data supporting the conclusions of this article will be made available by the authors, without undue reservation.
