# Supplementary material for: Perivascular spaces and basilar artery remodeling in Fabry disease—a dual vascular pathology
Source: Front Neurol. 2025 Dec 15;16:1689057. doi: 10.3389/fneur.2025.1689057 (PMC12745238; doi:10.3389/fneur.2025.1689057)
Supplement: Supplementary file 1 [file Table_1.DOCX]

**Supplementary Table 1. Demographic and clinical characteristics of the 26 study participants**

| **No** | **Sex** | **Age** | **Type** | **Age (Dx)** | **Duration (Tx), years** | **Mutation** | **Enzyme level** |  | **Pain** | **Ocular symptoms** | **Skin lesions** | **Stroke** | **LVMI** | **LVW thickness, mm** |
| --- | --- | --- | --- | --- | --- | --- | --- | --- | --- | --- | --- | --- | --- | --- |
| 1 | M | 19 | C | 12 | 9 | G271C | 2.10 |  | Yes | Yes | Yes | No | 99 | 9.3 |
| 2 | M | 22 | C | 19 | 3 | R227Q | 0.28 |  | Yes | Yes | Yes | No | 93.7 | 9.3 |
| 3 | F | 23 | C | 16 | 11 | R220* | 0.32 |  | Yes | Yes | No | No | 143 | 7.9 |
| 4 | F | 23 | C | 16 | 8 | P205L | 68.40 |  | Yes | Yes | Yes | No | 69.9 | 7.6 |
| 5 | F | 24 | C | 19 | 7 | L19P | NA |  | Yes | Yes | Yes | No | 57.5 | 7.3 |
| 6 | F | 32 | C | 27 | 6 | W287* | 32.8 |  | Yes | Yes | Yes | No | 70.4 | 9 |
| 7 | F | 36 | C | 34 | 4 | W226G | 0.12 |  | Yes | Yes | Yes | No | 76.3 | 8 |
| 8 | F | 36 | C | 29 | 9 | G261Vfsx8 | 0.28 |  | Yes | Yes | Yes | No | 79.7 | 8.8 |
| 9 | M | 37 | C | 30 | 6 | R220* | 0.30 |  | Yes | Yes | Yes | No | 121 | 11 |
| 10 | M | 39 | C | 32 | 9 | G261Vfsx8 | 0.06 |  | Yes | Yes | Yes | No | 80 | 10.5 |
| 11 | F | 41 | C | 40 | No | CIH | 84.40 |  | Yes | N/D | No | No | 106.5 | 9.8 |
| 12 | M | 70 | C | 70 | No | R301Q | 5.90 |  | Yes | No | Yes | No | NA | 8.5 |
| 13 | F | 24 | N | 24 | No | I91T | 28.40 |  | No | No | Yes | No | 69 | 9.29 |
| 14 | F | 25 | N | 24 | 1 | CIH | 0.32 |  | No | N/D | Yes | No | 51.9 | 6.9 |
| 15 | F | 31 | N | 30 | No | I91T | 28.80 |  | No | Yes | No | No | 78 | 7.8 |
| 16 | F | 43 | N | 18 | 1 | G271C | NA |  | No | Yes | No | No | 99 | 12.1 |
| 17 | F | 51 | N | 43 | No | R301Q | NA |  | No | N/D | Yes | No | NA | 12.1 |
| 18 | M | 52 | N | 49 | 9 | L19P | 3.20 |  | No | Yes | Yes | No | 188 | 14 |
| 19 | F | 56 | N | 55 | No | E66Q | NA |  | No | N/D | Yes | No | 70 | 9.3 |
| 20 | M | 56 | N | 57 | No | CIH | 0.44 |  | No | N/D | Yes | No | 58.38 | 6.6 |
| 21 | F | 56 | N | 51 | 9 | I91T | 6.00 |  | No | No | No | No | 156 | 13.2 |
| 22 | F | 60 | N | 59 | No | I91T | 12.70 |  | No | N/D | No | No | 119 | 9.6 |
| 23 | F | 60 | N | 54 | 9 | G261Vfsx8 | 0.40 |  | No | Yes | Yes | Yes | 167 | 15.3 |
| 24 | F | 61 | N | 54 | 9 | R220* | N/A |  | No | Yes | No | No | 88 | 8 |
| 25 | F | 62 | N | 56 | 8 | R220* | 4.60 |  | No | Yes | No | No | 179 | 16.7 |
| 26 | F | 68 | N | 67 | 1 | c.640-11T>A | 31.2 |  | No | Yes | No | No | 119.9 | 11.1 |

C, classic; CIH, complex intronic haplotype; Dx, diagnosis; Enz., enzyme; F, female; LVMI, left ventricular mass index; LVW, left ventricular wall; M, male; N, non-classic; N/A, not applicable; N/D, not done; Tx, treatment.

**Supplementary Table 2. Interaction between FD status and macrovascular/microvascular imaging indices for the association with the FD-related key marker (PVS burden) identified in the first-stage analysis (primary-adjusted model).**

| **Variable** | **p for interaction** | **q (FDR)** |
| --- | --- | --- |
| Diameter of ICA, Rt | 0.7662 | 0.9721 |
| Diameter of ICA, Lt | 0.9721 | 0.9721 |
| Diameter of BA | 0.3751 | 0.9721 |
| BATI | 0.6444 | 0.9721 |
| BADI | 0.8275 | 0.9721 |
| Fazekas, sum | 0.9665 | 0.9721 |
| Cerebral atrophy | 0.9292 | 0.9721 |

Pooled linear regression, including a multiplicative interaction term (FD vs. Control × predictor). Slopes correspond to the change in outcome per 1 SD increase in the predictor within each group. The models were adjusted for age, sex, and vascular risk factors. Benjamini–Hochberg false discovery rate (FDR) (q) is reported for the interaction term.

FD, Fabry disease; PVS, perivascular spaces; BADI, basilar artery diameter index; BATI, basilar artery tortuosity index; FDR, false discovery rate. Interaction p-values test the equality of slopes between the FD and Control groups.
